# Supplementary material for: Patterns of Intron Gain and Loss in Fungi
Source: PLoS Biol. 2004 Nov 30;2(12):e422. doi: 10.1371/journal.pbio.0020422 (PMC532390; doi:10.1371/journal.pbio.0020422)
Supplement: Table S1 — Also available at http://genes.mit.edu/NielsenEtAl/. (4.3 MB ZIP). [file pbio.0020422.st001.zip › NielsenEtAl/html/730.html]

AN0044.1.NCU09516.1.MG05032.1.FG02724.1


```
 CLUSTAL W (1.82) Multiple Sequence Alignments - Introns Inserted


Sequence 1: NCU09516.1	1175 aa
Sequence 2: FG02724.1	1154 aa
Sequence 3: MG05032.1	1166 aa
Sequence 4: AN0044.1	1202 aa
Alignment Length: 1291 aa
Number Identitical Residues: 453 aa
Alignment Score (without introns) 23570


MG05032.1 	----------MAPPIALETP------YYDQMDLSNGSEPPSKKRRFFSDSDDEAATQSRR
NCU09516.1	MDFTNEPLSKKRRFLGDQGDSDHVAGGPSSSPQFSAPPSSPPRKKLLQDPNSEVQPRVSK
FG02724.1 	----------MEQPLGDMS--------PWHPDNHMDPEPPTKRRRFLADPDEASSDPVAH
AN0044.1  	----------MNFDASNDRP-------LKKRRFFVDDPLDTAVTPAEKSPALDASSSAST
          	               .                        .       ..          

MG05032.1 	PSPPSPEIQ-------------HRQDTLDTPPTLSNHSAALGEPLNTITAQ---------
NCU09516.1	DADHNDDDDDDDDDDDEERPRFFTDDGTLTPHATKICAPWLNDMPKPDPRKGYLLKDVDT
FG02724.1 	DSSPLPPSK-----------RFFENEDETVPKTERLET--IDEKPNNASPK---------
AN0044.1  	HTDPNYSAN-------------GSPAQIQTQQEYNFSNGGHATQPATAAGTR--------
          	 :      .                    .                  .           

MG05032.1 	-----ETSRPEASLEDISGFDQATFEAVVGVEVKPDILAIIRQQCGSDLERAVNMYFDGT
NCU09516.1	PIATPRDVAPPVVESPQLAFDKDTFEAFVGEKVASDILHVISKNCGNNIERAVNMYLDGT
FG02724.1 	---SSSTELPTSQQEPSPSFDQETFETFIGDKVSEDVLSAIRSNCGNNIERAVNMYFDGT
AN0044.1  	-----THQAQSPAHDTLSDFDTEAFVSIVGEQVSPETLSQIRKLSDGSLEKAINVYFDGS
          	              .    **  :* :.:* :*  : *  * . ....:*:*:*:*:**:

MG05032.1 	WKNYKLKPKQ--------------KPTTITSSIRLNGGDGAKID-QVTSRPAIRPGAPGS
NCU09516.1	WKKLHRAPPVRVNSHSPLVVGGQSPKKSSTSQARSRSHAQAQPQPQSNTPTKVLPSMPDA
FG02724.1 	YKKFMKKSTR------------PAPPRPAASSSRTPNVSGER-----TIPIQTSKRMPNE
AN0044.1  	WKNAGSPGSSQTTLLSCERN--ASRPSPLHTSREQGTGPAENGADSVTEPISRSKLQPAR
          	:*:         .  :       :   .  :. .       .   . .         *  

MG05032.1 	RYVGAFGVEGWATRSGTNLLKHGDVVRIERQKIQPPKPPTKGKGATNKLGAAVGGLRVSA
NCU09516.1	RYVGAFGVEGWATRSGTGLLRHGDSVKIERQKIQPPTVARKGQ---TKPGTPQSIPRVSA
FG02724.1 	RYIGAFGVEGWATRSGTNLLKHGDIVKIERQKRAPPPTKSKGK---AGPVTPS----RGF
AN0044.1  	RYLGAFGVEAWATRSGIGLIKHGDTVNIERARSQPLSTRGRTG---------------KL
          	**:******.****** .*::*** *.*** :  *     :                   

MG05032.1 	AAARRVDVIVRFTDRQGTEIGRLAKDTANWVSTLIDQKVCKFEGTCVYAPERLRTNDTVF
NCU09516.1	AAAKRVDVIVRFNDASGRELGRLAKDTANWVSTLIDQNICRFEGICVYAPERLRTNETVF
FG02724.1 	GAPRRQDVVVRFTTQSGTEVGRLAREAANWVSALIDEKICRFEGTVVYAPERLRTNDTIF
AN0044.1  	RVNQKGDVLTRFTNTAGQEIGRLPRETAEWVSTLLDQKICEFRGVCVFAPDRLRVNDTIY
          	 . :: **:.**.   * *:***.:::*:***:*:*:::*.*.*  *:**:***.*:*::

MG05032.1 	IQLRCSLLNEAFDTRRFQLS--DNRNTGMFEEQETAEERNLRLRQVALVRLFQEINLMPI
NCU09516.1	LQLKCYMLRSAFLGRTLQLA--DNRAAGFHEKDETTEEKDLRLRQVALVRLFQEINIVPS
FG02724.1 	LQLRCSLLNSAFFSRPFQLA--DDRSAAFFNQNETNDEKTLRMRQVALVKLFQEINLHPT
AN0044.1  	LQLRCFMRIEAFQPKELPQKQDDNRATTIFEQEESAEEKQLRLRQVALVQLFDEIGLKST
          	:**:* :  .**  : :   ..*:* : :.:::*: :*: **:******:**:**.: . 

MG05032.1 	RVNEATAKNSRQGLLQAAEMDDQKEAQPQSTSVENSD-------SPQSEAEDGKELEQDQ
NCU09516.1	RGNAAAAKDARKDLLEAADSAEKKAMDKAKAGDHNTN----GLASPPEEAEEGQELEQDQ
FG02724.1 	LTN-SATKDGRKGLLQAAEQDEEKQKEVKKSDGNGTNNTKEANSSQSSDTEDGEELEQDQ
AN0044.1  	TQDDEIKKQRKEGLLRAAEMADQEAKKLAKSGNTDSG------------DEEPAELEQDQ
          	  :    *: ::.**.**:  :::  .  .:.  .:.             *:  ******

MG05032.1 	LDALYKKAQSFDFDTPEAEPAETFAMDLRPYQKQSLYWMLAKEKDEAGKDRESSIHPLWE
NCU09516.1	LDALYKKAQSFDFSTPEAEPANTFAMTLRPYQKQSLYWMLAKEKNQRTEDRETSMHPLWE
FG02724.1 	LDALYKKAQSFDFNTPEAEPADTFAMTLRKYQKQALHWMMAKEKDEKS-HREPLMHPLWE
AN0044.1  	LDALYKKAQSFDFSMPEAQPPSSFAMDLRKYQKQALYWMLSKEKDKKS-GREVSIHPLWE
          	*************. ***:*..:*** ** ****:*:**::***::    **  :*****

MG05032.1 	E~YQWPTKDFDDQDVPQVADQPSFYVNPYSGEMSLEFPAQEQHCLGGILADEMGLGKTIQ
NCU09516.1	D2--------------------------------------SQSCIT---------GKTIQ
FG02724.1 	Q~YEWPLKDVDENDLPQIEGQSKFYVNPYSGDLSLDFPVQEQHCLGGILADEMGLGKTIQ
AN0044.1  	E~YDWPLKDVDDKDLPIIEGINHFYVNPYSGELSLDFPAQEQHCLGGILADEMGLGKTIE
          	:  . . .. .... .   .      .. :.. : . . ..* *: .  :.. . ****:

MG05032.1 	MLSLIHTHKPHAAAAADA---TALTVNDLQRMPGGG--NKVQPAPYTTLVVAPMSLLSQW
NCU09516.1	MLSLIHSHRSEVAIKARE--AGPTSVNNLPRLPTVSGQKTTIDAPCTTLVVAPMSLLAQW
FG02724.1 	MLSLVHTHRSEVALEARQSVVARSNVNQLTRLGKNS--ESILDAPCTTLVVAPMSLLSQW
AN0044.1  	MLSLVHSHRNLPPTQSLG---------NLTRLPVSG----VVPAPYTTLVVAPMSLLAQW
          	****:*:*:   .  :           :* *:   .       ** ***********:**

MG05032.1 	QSEAENASKEGTLKSIVYYGNDKHANLQALC--SNPATAPDVIITSYGIVLSEFGQIAGS
NCU09516.1	QSEAENASKEGTFKTMMYYGAEKNVDLVTMCCEANAANAPDVIITSYGVVLSEFTQLA-T
FG02724.1 	QSEAEKASKDGTMKTELYYGNEKSSNLQALCCASNAANAPDLVITSYGVVLSEFSSLA-A
AN0044.1  	EGEALKASRNGSMKVLMYYGNEKNVNLREMCSAGNAA-APNMILTSYGVVMSEHRTHQAL
          	:.** :**::*::*  :*** :*  :*  :*. .*.* **::::****:*:**.    . 

MG05032.1 	KSAKRDGHTGLFSVNFLRVILDEAHNIKNRQAKTSKACYELSADHRWVLTGTPIVNRLED
NCU09516.1	KNGDRLSSRGLFSLNFFRVILDEAHNIKNRQAKTSRACYEIAAEHRWVLTGTPIVNRLED
FG02724.1 	RNGDKSFHNGLFSLRFFRIIIDEAHHIKNRSSKTSKACYEISATHRWALTGTPIVNKLED
AN0044.1  	APGTSWTPGNLFSVDFFRVILDEAHIIKNRRSKTARACYDLKATHRWVLTGTPIVNRLED
          	  .      .***: *:*:*:**** **** :**::***:: * ***.********:***

MG05032.1 	LFSLVRFLRVEPWNNFSFWRTFITVPFESKDFMRALDVVQTVLEPLVMRRTKDMKTPSGQ
NCU09516.1	LFSLVRFLRVEPWNNFSFWRTFITVPFESKNFVRALDVVQTVLEPLVMRRTKDMKTPDGQ
FG02724.1 	LFSLVRFLGVEPWNNFSFWRTFITVPFESGDFMRALDVVQTVLEPLVLRRTKDMKTPDGE
AN0044.1  	LFSLVRFLRVEPWNNFSFWKTFITAPFESKEVVRAISVVQTVLEPLVLRRTKSMKTPEGE
          	******** **********:****.**** :.:**:.**********:****.****.*:

MG05032.1 	P2LVALPPKTIEIVDVEFSKTERAVYDHIINRARSAFQKNVEAGTVMKAYTSIFAQILRL
NCU09516.1	F~LVPLPPKHIEIVDIELSEPERAVYDYVFNRAKRTLFDNMQAGTVMKAFTSIFAQILRL
FG02724.1 	P~LVLLPPKQIEIVNVELSETERDVYNYIFNKAKRTFSQNVEAGTVMKAFTTIFAQILRL
AN0044.1  	P~LVPLPKRTIRIEKVELIEQEREIYNHIYTRAKQTFNSNVAAGTLLKSYSTIFAQLLRL
          	  ** ** : *.* .:*: : ** :*::: .:*: :: .*: ***::*::::****:***

MG05032.1 	RQSCCHPVLVRNQDIVADEDEAAAAADAVAGLADDMDLHSLIERFTASTDDPADANA---
NCU09516.1	RQSCCHPVLVRNQEILADEEEANMAADVAAGLADDMDLQTLIERFTATTDDASKTNN---
FG02724.1 	RQSCCHPILVRNRDIVADEVEAGAAADAAAGLADDMDLESLITSFTAVTDKASKESNQ--
AN0044.1  	RQTCCHPILTRNKAIVADEEDAAAAADQDSDLKDDMDLQELINRFTATTSDAESSNEPPD
          	**:****:*.**: *:*** :*  ***  :.* *****. **  *** *... . .  ..

MG05032.1 	----FGAHVLSQIRDEAANECPICTEEPMIEQTVTGCWHSTCKKCILDYIKHQTDRHEVP
NCU09516.1	---NFGAHVLRQIRDEAVNECPICAEEPMIDQAVTGCWHSACKKCLLDYIKHQTDRNEVP
FG02724.1 	---TFGAHALEQIRDEAENECPLCFEEPMNDQTVTGCWHSACKKCLLDYIKHQTDKAEVP
AN0044.1  	PSMKFTAHALRQIQTESAGECPICSEEPMIDPAVTACWHSACKGCLKDYIQHQRDKGVQP
          	.: .* **.* **: *: .***:* **** : :**.****:** *: ***:** *:   *

MG05032.1 	RCVSCRQPINERDLFEVVRHDNDVYDDDEDKPG-----SVFKQKQPDQPRRISLQRVGVN
NCU09516.1	RCFQCREHINIRDIFEVIRHDDDLE--------------TSSTPGASPEPRISLQRVGAN
FG02724.1 	RCFSCREPINKRDLFEVVRHDDDSD--------------MMSK-----KPRISLQRVGVN
AN0044.1  	RCFSCRADLNPQDIFEVVRYQSPNTTPTEQTPSSIGGDNVYSSSQPPPPPRISLRRINPL
          	**..**  :* :*:***:*::.      .....: ....  .   .    ****:*:.  

MG05032.1 	DSSTKVVALIQHLRDLRRERPR-AKVVVFSQFTSFLTLIEGSLDRANMHHVRLDGTMAQK
NCU09516.1	DSSAKIVALISHLRTLRQEHPK-MKSLVISQFTSFLSLISSALTRHKISFLRLDGSMSQK
FG02724.1 	ASSAKVVALMSELRALRREHPK-MKSVVFSQFTSFLSLIEPALTRANIKFLRLDGSMAQK
AN0044.1  	SPSAHTSAKIHALLAHLVRVPAGTKSVVFSQFTSFLDLIGPQLTKAGISFVRLDGTMAQK
          	 .*::  * :  *     . * . * :*:******* **   * :  : .:****:*:**

MG05032.1 	TRVAVLEEFKACSKFT---------------------------------VFLISLRAGGV
NCU09516.1	ARAAVLTEFQSTNKFC---------------------------------VLLLSLKAGGV
FG02724.1 	ARAAVLNEFTEKKGFT---------------------------------ILLLSLRAGGV
AN0044.1  	ARAEVLAQFTKFETFTQEELDQAESTSAPSGLTPTPKTPKQSSSPSSPTVLLISLKAGGV
          	:*. ** :*   . * ... ..:.::::.:. :.:..:...:::.::.:::*:**:****

MG05032.1 	GLNLTEASRVYMCDPWWSFSVESQAIDRVHRMGQSEEVKVYRFIVKNSVEERMLKIQDRK
NCU09516.1	GLNLTSAKRVYMMDPWWSFAVEAQAIDRVHRMGQEDEVRVYRFIVKQSVEMRMLRVQERK
FG02724.1 	GLNLTSAGRVFMMDPWWSFAVEAQAIDRVHRMGQEAEVQVKRFVVKESVEERMLKVQERK
AN0044.1  	GLNLTAASNVFMMDPWWSFAIEAQAIDRVHRMGQLRDVNVVRFIVKDSIEERMLRVQERK
          	***** * .*:* ******::*:***********  :*.* **:**:*:* ***::*:**

MG05032.1 	KFI2ATSLGMMSD---DEKRLARIEDIKELLS--
NCU09516.1	KFI2ATSLGMMSD---EEKKMQRIEDIKELLSSD
FG02724.1 	KFI2ATSLGMMND---EEKKLQRIEDIKELLS--
AN0044.1  	MGI~AGSLGLMGEGNEEERRKERIEELRLLFE--
          	  * * ***:*.:...:*::  ***::: *:.
```
